# Combined effects of carotid plaques and hypertension on the risk of cardiovascular disease and all‐cause mortality

**DOI:** 10.1002/clc.23372

**Published:** 2020-05-12

**Authors:** Wen Li, Jianqiu Zhao, Lu Song, Shuohua Chen, Xuemei Liu, Shouling Wu

**Affiliations:** ^1^ Department of Ultrasound Shanghai Sixth People's Eastern Hospital Shanghai China; ^2^ Department of Anesthesiology Anting Hospital Shanghai China; ^3^ Department of Cardiology Kailuan General Hospital Affiliated to North China University of Science and Technology Tangshan China; ^4^ Department of Ultrasound Beijing Hospital of Traditional Chinese Medicine Affiliated to the Capital Medical University Beijing China

**Keywords:** all‐cause mortality, cardiovascular disease, carotid plaque, hypertension

## Abstract

**Background:**

Both hypertension and atherosclerotic plaques are risk factors for cardiovascular disease (CVD).

**Hypothesis:**

This study aimed to investigate whether the combined effects of carotid plaques and hypertension increase the risks of CVD and all‐cause mortality.

**Methods:**

Patients from the stroke and elderly cohorts of the Kailuan study in China who completed a carotid sonography examination were included in the study. Participants in both cohorts underwent physical examinations between 2010 and 2011 and were divided into four groups: no carotid plaques with normal blood pressure (n = 2227), hypertension only (n = 1290), carotid plaques only (n = 1128), and hypertension with carotid plaques (n = 1862). The outcomes included the first occurrence of CVD and all‐cause mortality.

**Results:**

Among the 6507 participants (mean age, 58.1 ± 11.8 years, 61% males), 157 cardiovascular events, and 210 deaths occurred after average follow‐ups of 4.5 and 4.9 years, respectively. After adjusting for covariates, carotid plaques only and hypertension with carotid plaques were associated with excess risk (hazard ratio [HR]; confidence interval [CI]) for the first occurrence of CVD (HR = 1.85; 95% CI, 1.01‐3.44; and HR = 2.97; 95% CI, 1.66‐5.29, respectively), cerebral infarction (HR = 2.66; 95% CI, 1.16‐6.15; and HR = 4.15; 95% CI, 1.87‐9.19, respectively), and all‐cause mortality (HR = 1.96; 95% CI, 1.16‐3.31; and HR = 1.85; 95% CI, 1.09‐3.13, respectively).

**Conclusions:**

The combination of hypertension and atherosclerotic plaques may increase the risk of CVD events and all‐cause mortality, especially cerebral infarction, compared with participants without those factors.

## 1 INTRODUCTION

Cardiovascular diseases (CVDs) encompass a number of vascular conditions such as coronary heart disease (CHD), cerebrovascular disease, peripheral artery disease, rheumatic heart disease, congenital heart disease, deep vein thrombosis, and pulmonary embolism.^1^ CVDs are the leading causes of death worldwide, with an estimated 17.9 million deaths in 2016, or 31% of all global deaths.^1^ One of every three deaths in the United States is attributable to CVDs,^2^, ^3^ and the prevalence of CVDs in China is continuously increasing because of changes in lifestyle, urbanization, and the aging population.^4^ Currently, one in five Chinese adults are afflicted with CVDs, which account for >40% of deaths from all causes in China.^5^ Therefore, the burden of CVDs is substantial and has become a serious public health issue. Most CVDs can be prevented using proper lifestyle interventions.^1^

Hypertension is a primary risk factor for CVD and all‐cause mortality.^6^, ^7^, ^8^ People with hypertension (blood pressure ≥140/90 mm Hg or treatment with antihypertensive medications) have a significant lifetime risk of overall CVD by 30 years of age.^9^ Given a systolic blood pressure (SBP) of 115 mm Hg and diastolic blood pressure (DBP) of 75 mm Hg, each 20 mm Hg increase in SBP (or an equivalent of a 10 mm Hg DBP increase) is associated with more than a twofold risk of CVD and death.^10^ More than half of all cases of CVDs are associated with hypertension in China,^4^ and the risk of all‐cause mortality from CHD and stroke is expected to increase from 14% to 95% and 290% among individuals with hypertension.^11^

Atherosclerosis is the main cause of many CVDs. The formation of an atherosclerotic plaque is the hallmark of atherosclerosis, and vascular occlusion caused by plaque rupture is an important pathophysiological mechanism in the development of ischemic CVD.^12^ The risk of CVD among individuals with atherosclerotic plaques is 1.3 to 2.8 times higher than that of individuals without plaques, as reported by several large cohort studies.^13^, ^14^, ^15^, ^16^ Therefore, the formation of atherosclerotic plaques is a major risk factor for the development of CVD. The 2016 European Guidelines on CVD Prevention in Clinical Practice recommended the use of carotid artery scanning for atherosclerotic plaque screening as part of CVD risk assessments.^17^ After the addition of atherosclerotic plaques to the Framingham Risk Factor Model, the ability to predict CVD events increased by 2% to 5%, which was reported in several large cohort studies.^18^ Therefore, the presence of atherosclerotic plaque has become an important measure of the level of risk in clinical studies.

In theory, because hypertension and atherosclerotic plaques are both independent risk factors for CVD, patients with these two factors may be at even higher risk for CVD events compared with patients with only one of these factors, but there are no scientific reports or empirical data on the effects of the relationships among hypertension, atherosclerotic plaques, and CVD, and their effects on all‐cause mortality. To address this knowledge gap, data collected as part of the Kailuan study were analyzed to examine whether the combined risk factors of hypertension and atherosclerotic plaques could be associated with an increase in CVD events and all‐cause mortality.

## 2 METHODS

### 2.1 Study design and population

The study population consisted of the stroke and elderly cohorts from the Kailuan cohort study, an ongoing prospective study, which has been described previously.^19^ The study was initiated in 2006 by the Kailuan (Group) Co., Ltd. in Tangshan City, a large littoral metropolis located 180 km southeast of Beijing.

Regarding the stroke cohort, 5440 participants in the Kailuan study who underwent physical examinations between June 2010 and June 2011 were selected using stratified random sampling. The inclusion criteria were: (a) age ≥40 years; (b) no history of stroke, transient ischemic attack, or coronary disease at baseline, as assessed using a validated questionnaire and by experienced physicians; and (c) written informed consent. The exclusion criteria were: (a) serious physical disability or inability to undergo physical check‐ups/examinations; or (b) a history of ischemic stroke or transient ischemic attacks, excluding lacunar infarction. The final number of participants who were included in the stroke cohort and who completed the carotid sonography examination was 5425.

**FIGURE 1:**
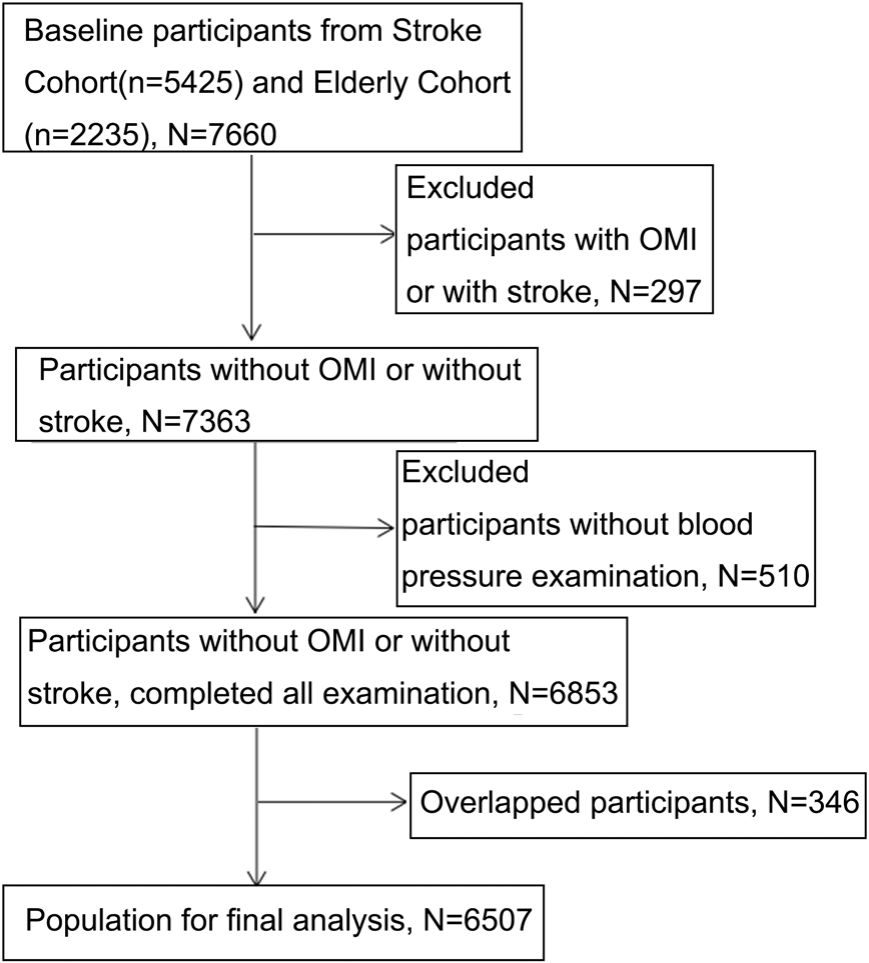
Flowchart of the study participants

Regarding the elderly cohort, after the third Kailuan physical examination in 2010 to 2011, cluster sampling of the patients with physical examinations was conducted at the Kailuan General Hospital, Kailuan Linxi Hospital, and Kailuan Zhaogezhuang Hospital. Retired staff of the Kailuan Group, age ≥60 years, served as an alternative population, comprising 25% of the 3064 participants. Of the 2464 participants included in the study cohort, 2235 completed the carotid sonography examination, and 346 participants in the two groups were from overlapping populations. The exclusion criteria were: (a) a history of stroke or myocardial infarction (MI) (n = 297); or (b) the absence of blood pressure data at baseline (n = 510), leaving 6507 participants in the final statistical analysis (Figure 1).

This study was conducted in accordance with the guidelines of the Helsinki Declaration and was approved by the ethics committees of the Kailuan General Hospital ([2006]Approval No.5) and the Beijing Tiantan Hospital (Approval No.:2010‐001‐02).

### 2.2 Data collection

Data on demographic characteristics, including age, sex, household income, education, lifestyle, and related variables, were collected using standardized questionnaires by trained investigators. Smoking was recorded as “yes” or “no” based on participants' self‐reports. Drinking was defined as alcohol consumption ≥100 mL/day for more than 1 year. Hypertension was defined as the presence or history of hypertension or the use of antihypertensive medication or a SBP ≥140 mm Hg or a DBP ≥90 mm Hg. Diabetes mellitus was defined as the presence or history of diabetes mellitus or current treatment with insulin or oral hypoglycemic agents or a fasting blood glucose (FBG) level ≥126 mg/dL. Dyslipidemia was defined as the presence or history of dyslipidemia, or the current use of lipid‐lowering medication, or total cholesterol (TC) ≥220 mg/dL, triglyceride ≥150 mg/dL, or low‐density lipoprotein cholesterol (LDL‐C) ≥140 mg/dL.

### 2.3 Assessment of cranial‐carotid plaques

All study participants underwent a bilateral carotid duplex ultrasound (Philips iU‐22 Ultrasound System, Philips Medical Systems, Bothell, Washington) to determine the presence of carotid plaques. Carotid artery plaque is defined as a focal structure that encroaches into the arterial lumen at least 0.5 mm or 50% of the surrounding IMT value or demonstrates a thickness >1.5 mm, as measured from the media‐adventitia interface to the intima‐lumen interface.^20^

### 2.4 Follow‐up and outcomes

Participants were followed using face‐to‐face interviews during routine medical examinations every 2 years until December 31, 2015, the occurrence of an event of interest, or death. The outcome information for patients who could not complete the face‐to‐face follow‐up interviews was obtained by checking medical records from the hospital and the patient's medical insurance. The study endpoints included the first occurrence of a CVD event (stroke or MI) and all‐cause mortality. The diagnosis of stroke was confirmed by brain‐computed tomography (CT) or magnetic resonance imaging (MRI) and classified into two main types: cerebral infarction and hemorrhagic stroke. MI was defined in accordance with the 2007 universal definition.^21^ Deaths were confirmed by checking the death certificates from the provincial vital statistics offices. All outcomes were approved by the Data Safety Monitoring Board and the Arbitration Committee for Clinical Outcomes.

### 2.5 Statistical analyses

Continuous variables were reported as means ± standard deviations, and compared using analysis of variance with the SNK or Dunnett T3 post hoc test. Categorical variables were reported as percentages, and analyzed using the chi‐square test. Cox proportional hazards regression was used to estimate the risks of events by calculating the hazard ratios (HR) and 95% confidence intervals (CI). Other relevant risk factors were adjusted in the regression analysis. The rates of events were estimated using the Kaplan‐Meier method and compared across groups using the log‐rank test. All statistical tests were two‐tailed, and the significance level was set to 0.05. Statistical analyses were performed using SAS 9.3 (SAS Institute, Cary, North Carolina).

### 2.6 Clinical trial registration

Chinese Clinical Trials Registry, ChiCTR‐TNC‐11001489 **(**retrospective registration).

## 3 RESULTS

### 3.1 Baseline characteristics of the study population

The data from 6507 participants (mean age, 58.1 ± 11.8 years, 61% of males) were analyzed. The baseline characteristics of the entire cohort are presented in Table 1. Significant differences among the groups were found for age, sex, smoking habits, drinking habits, FBG, TC, LDL‐C levels, carotid intima‐media thickness, and follow‐up.

**TABLE 1:** Baseline characteristics of the study population

Characteristics	No carotid plaque and normal blood pressure	Hypertension only	Carotid plaque only	Hypertension with carotid plaque	All
Age (y)	50.6 ± 8.7	54.6 ± 10.1^a^	61.6 ± 11.8^ab^	64.6 ± 11.2^abc^	57.3 ± 11.9
Male (%)	979 (44.0)	795 (61.6)^a^	792 (70.2)^ab^	1403 (75.3)^abc^	3969 (61)
Smoking (%)	595 (26.7)	472 (36.6)^a^	496 (44)^ab^	758 (40.7)^abc^	2321 (35.7)
Drinking (%)	553 (24.8)	484 (37.5)^a^	367 (32.5)^ab^	745 (40.0)^ac^	2149 (33.0)
Heart rate (bpm)	69.5 ± 9.7	72.5 ± 10.6^a^	69.7 ± 10.5^b^	72.4 ± 12.3^ac^	70.9 ± 10.9
FBG (mmol/L)	5.34 ± 1.15	5.63 ± 1.33^a^	5.68 ± 1.69^a^	5.96 ± 1.85^abc^	5.64 ± 1.53
Systolic BP (mm Hg)	117.2 ± 11.5	142.7 ± 15.9^a^	121.4 ± 10.7^ab^	149.2 ± 17.8^abc^	132.2 ± 20.2
Diastolic BP (mm Hg)	76.7 ± 6.9	91.8 ± 9.6^a^	76.6 ± 7.2^b^	88.4 ± 10.9^ab^	83.1 ± 11.0
BMI (kg/m^2^)	24.3 ± 3.0	26.1 ± 3.4^a^	24.1 ± 3.1^b^	25.5 ± 3.3^abc^	25.0 ± 3.3
TC (mmol/L)	4.90 ± 0.89	5.00 ± 1.01^a^	5.24 ± 1.68^ab^	5.29 ± 1.92^ab^	5.10 ± 1.43
LDL‐C (mmol/L)	2.58 ± 0.71	2.69 ± 0.78^a^	2.72 ± 0.78^a^	2.78 ± 0.88^abc^	2.67 ± 0.79
HDL‐C (mmol/L)	1.67 ± 0.48	1.57 ± 0.41^a^	1.61 ± 0.45^ab^	1.57 ± 0.45^ac^	1.61 ± 0.45
Taking hypoglycemic drug (%)	44 (2.0)	63 (4.9)^a^	70 (6.2)^ab^	172 (9.2)^abc^	349 (5.4)
Carotid intima‐media thickness	0.76 ± 0.13	0.81 ± 0.15^a^	0.91 ± 0.18^ab^	0.97 ± 0.19^abc^	0.86 ± 0.19
Follow‐up (y)	5.1 ± 0.4	5.0 ± 0.4	4.9 ± 0.6	4.8 ± 0.8	4.9 ± 0.6

Abbreviations: BMI, body mass index; BP, blood pressure; FBG, fasting blood glucose; HDL‐C, high‐density lipoprotein cholesterol; LDL‐C, low‐density lipoprotein cholesterol; TC, total cholesterol. a, compared with the first group, *P* < 0.05; b, compared with second group, *P* < 0.05; c, compared with the third group, *P* < 0.05.

### 3.2 CVD events and all‐cause mortality

After a mean follow‐up of 4.5 ± 0.7 years, 157 CVD events occurred. Among the overall study population, 44 participants had MIs, and 113 had strokes, including 105 cerebral ischemic strokes, and 8 cerebral hemorrhagic strokes. The incidence of CVDs was 53.91 per 10 000 person years. A total of 210 deaths occurred after a mean follow‐up of 4.9 ± 0.6 years. The incidence of all‐cause mortality was 65.54 per 10 000 person years (Table 2).

**TABLE 2:** Incidence rate of adverse outcomes

	All‐cause mortality	CVD	Stroke	Cerebral hemorrhage	Cerebral ischemic	MI
*No carotid plaque and normal blood pressure*						
Number of events	22	19	11	2	9	8
Cumulative incidence (95% CI)	1.0 (0.58‐1.42)	0.9 (0.50‐1.30)	0.5 (0.21‐0.79)	0.1 (−0.04‐0.24)	0.4 (0.14‐0.66)	0.4 (0.12‐0.68)
Per 10 000 person years	19.54	18.53	9.75	1.94	8.75	7.78
*Hypertension only*						
Number of events	24	24	17	1	16	7
Cumulative incidence (95% CI)	1.9 (1.15‐2.65)	1.9 (1.15–2.65)	1.3 (0.69‐1.91)	0.1 (−0.01‐0.3)	1.2 (0.62‐1.78)	0.5 (0.13‐0.87)
Per 10 000 person years	37.49	41.68	26.53	1.72	27.68	12.07
*Carotid plaque only*						
Number of events	56	26	20	2	18	6
Cumulative incidence (95% CI)	5.0 (3.72‐6.28)	2.3 (1.43‐3.17)	1.8 (1.02‐2.58)	0.2 (−0.08‐0.48)	1.6 (0.87‐2.33)	0.5 (0.1‐0.9)
Per 10 000 person years	102.12	51.74	36.06	3.92	35.66	11.81
*Hypertension with carotid plaque*						
Number of events	108	88	65	3	62	23
Cumulative incidence (95% CI)	5.8 (4.74‐6.86)	4.7 (3.74‐5.66)	3.5 (2.67‐4.33)	0.2 (−0.03‐0.43)	3.3 (2.49‐4.11)	1.2 (0.71‐1.69)
Per 10 000 person years	121.42	108.87	73.33	3.62	76.08	27.90
*All*						
Number of events	210	157	113	8	105	44
Cumulative incidence (95% CI)	3.2 (2.77‐3.63)	2.4 (2.03‐2.77)	1.8 (1.47‐2.13)	0.1 (0.03‐0.17)	1.6 (1.30‐1.90)	0.7 (0.49‐0.91)
Per 10 000 person years	65.54	53.91	35.36	2.71	35.89	14.96

Abbreviations: CI, confidence interval; CVD, cardiovascular disease; MI, myocardial infarction.

### 3.3 HRs for CVD and all‐cause mortality by group

The results of the multivariable Cox proportional hazards regression analysis are shown in Table 3. After adjusting for all covariates, the hypertension only group did not show an increased risk for all‐cause mortality or CVD, but the risk of cerebral ischemic stroke was 2.25 times higher (95% CI, 1.01‐5.24). The groups with carotid plaques only and hypertension with carotid plaques were both associated with excess risks for the occurrence of CVD (HR 1.85; 95% CI, 1.01‐3.44; and HR, 2.97; 95% CI, 1.66‐5.29, respectively), cerebral infarction (HR, 2.66; 95% CI, 1.16‐6.15; and HR, 4.15; 95% CI, 1.87‐9.19, respectively), and all‐cause mortality (HR, 1.96; 95% CI, 1.16‐3.31; and HR, 1.85; 95% CI, 1.09‐3.13, respectively).

**TABLE 3:** Multivariable Cox regression analysis for adverse outcomes

Variables	CVD		Cerebral infarction		All‐cause mortality	
	HR (95% CI)	*P*	HR (95% CI)	*P*	HR (95% CI)	*P*
No carotid plaque and normal blood pressure	1.00		1.00		1.00	
Hypertension only	1.59 (0.85‐2.98)	.144	2.25 (1.01–5.24)	.046	1.24 (0.68‐2.25)	.191
Carotid plaque only	1.85 (1.01‐3.44)	.042	2.66 (1.16‐6.15)	.012	1.96 (1.16‐3.31)	.019
Hypertension with carotid plaque	2.97 (1.66–5.29)	<.001	4.15 (1.87–9.19)	<.001	1.85 (1.09–3.13)	.022

*Note:* Models are adjusted for age, male sex, family history of diabetes, smoking, alcohol, BMI status, FBG, TC, LDL‐C, HDL‐C, and taking hypoglycemic drugs.

Abbreviations: BMI, body mass index; CVD, cardiovascular disease; FBG, fasting blood glucose; HDL‐C, high‐density lipoprotein cholesterol; LDL‐C, low‐density lipoprotein cholesterol; TC, total cholesterol.

### 3.4 Sensitivity analysis results

Sensitivity analysis was performed after removing the lipid‐lowering drug, and the results were consistent with the above results (Table S1).

### 3.5 Survival analysis

**FIGURE 2:**
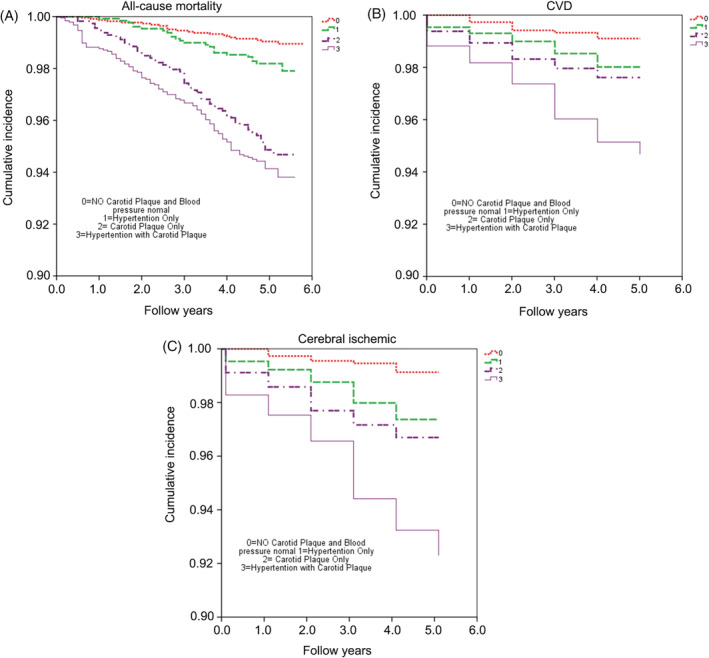
Survival Kaplan‐Meier analysis. A, All‐cause mortality. B, Cardiovascular diseases (CVDs). C, Cerebral ischemia

Figure 2 presents the survival analyses. Figure 2A shows that the carotid plaque only and hypertension with carotid plaque groups had lower survival than the normal and hypertension only groups. Figure 2B,C shows that the hypertension with carotid plaque group had a higher occurrence of CVD and stroke than the three other groups.

## 4 DISCUSSION

Both hypertension and atherosclerotic plaques are risk factors for CVD. This study aimed to investigate whether the combined effects of carotid plaques and hypertension increase the risks of CVD and all‐cause mortality. The results indicate that both hypertension and atherosclerotic plaques are risk factors for CVD. More importantly, we found that the combination of hypertension and atherosclerotic plaques further increased the risk for CVD events, especially cerebral infarction.

In the present study, hypertension increased the risk of cerebral ischemic stroke by 2.25 times. On the other hand, atherosclerotic plaques increased not only the risk for CVD and cerebral ischemic stroke but also the risk of all‐cause mortality. These data are similar to the results of studies by Northern^14^ and Kitamura et al^13^ that found that carotid plaques increased the risk of stroke by 2.1 and 3.0 times, respectively, in older adults. These findings might suggest that atherosclerotic plaques are associated with a higher risk of a vascular event than hypertension alone among middle‐aged and older adults. Therefore, those results strongly support the recommendation of the 2016 European Guidelines on CVD Prevention in Clinical Practice to include the examination of atherosclerotic plaques in all assessments of CVD risk.^17^

As shown in the results, hypertension and atherosclerotic plaques in combination increased the risk of CVD and all‐cause mortality, especially the risk of cerebral ischemic stroke by 4.15 times. Considering the effect of taking lipid‐lowering drugs on atherosclerotic plaques, we removed the lipid‐lowering drug in the sensitivity analysis, and the results were still consistent with the whole‐population analysis. In a study by Cuspidi et al,^22^ more than 50% of low‐moderate risk hypertensive patients showed an increased risk for CVD after the addition of carotid intima‐media thickness or plaques to the evaluation indices. When the presence or absence of plaques was added to the original prediction model, an additional 7.7% of the high‐risk individuals with CHD were identified, which enhanced the predictive value of atherosclerotic plaques for CHD in the Atherosclerosis Risk in Communities study,^23^ supporting the present study. This result is very important for Chinese people because the morbidity of strokes is three times higher than that of MIs in this population (7/2.5 million),^4^ which is much higher than Western countries, especially for cerebral ischemic stroke.^2^ Based on the present, we suggest that in addition to controlling blood pressure, actively adjusting lipid treatment may help stabilize or even eliminate plaques, and therefore, should be adopted to treat patients with combined hypertension and carotid plaques to minimize the residual risk of ischemic stroke. Indeed, it has been shown that statin treatment can lead to atherosclerotic plaque regression,^24^ highlighting the importance of blood lipid management.

Theoretically, both hypertension and carotid atherosclerosis are independent risk factors for CVD,^25^ but in fact, both hypertension and atherosclerosis are part of a complex cluster of metabolic disturbances that include hypertension, dyslipidemia, diabetes, obesity, low‐grade chronic inflammation, arteriosclerosis, atherosclerosis, and detrimental lifestyle habits.^26^, ^27^, ^28^ Among the patients with combined hypertension and carotid plaques, the risk of all‐cause mortality was increased (OR = 1.85), but was still somewhat lower than that of the patients with carotid plaques only (OR = 1.96). This result has two possible explanations. First, the groups with hypertension might have other diseases such as high cholesterol and diabetes when they visited the doctors in the early stage. Therefore, the effective treatment and control of blood glucose, blood lipids, blood pressure, and other risk factors can reduce mortality.^4^, ^29^, ^30^ Second, smoking can increase the risk of all‐cause mortality and deaths caused by CHD.^31^ The ratio of patients who smoked in the group with carotid plaques was higher than in the group with combined hypertension and carotid plaques (44% and 40.7%, respectively), which could have also increased the risk of all‐cause mortality. In addition, the group with hypertension alone only had a higher risk of stroke, without an effect of hypertension on mortality, which goes against the literature.^6^, ^7^, ^8^ Again, the presence of other comorbidities and their treatment might have reduced the mortality in those patients. Blood pressure was only measured once at the 2010 to 2011 examination, and no confirmation or monitoring of the change in time was performed. In addition, the concept of blood pressure as a risk factor depends upon the selection of a cut‐off point, which poses some controversies.^32^, ^33^, ^34^ Nevertheless, hypertension remains a strong risk factor for stroke,^6^, ^7^, ^8^ which was observed in the present study.

The findings of this study have important clinical significance. First, patients with hypertension continue to have a higher risk of CVD than those who do not have hypertension,^35^ and the morbidity of carotid plaque is very high among hypertensive patients.^36^ Although blood pressure may return to normal, carotid plaques will not fade away disappear so easily, at least not without a pharmacological intervention.^24^ Therefore, we speculate that carotid plaques could be a residual risk factor. Second, among 44.7% of Chinese adults (35‐75 years old) with hypertension, blood pressure was reduced to normal in 7.2% of them,^37^ at the same time, the achieved rate of dyslipidemia among Chinese adults was reported to be only 39.7%.^38^ The high prevalence and low success rates of treatment may be among the reasons for the high incidence of cerebral stroke. Therefore, patients with hypertension should be screened routinely for the presence of carotid plaques, and actively treated with lipid‐lowering medications. The earlier treatment is initiated, the more likely it will be successful in reducing the burden of CVD.

Our research has some limitations. First, we measured only the presence or absence of carotid plaques (a dichotomous variable) as the index of risk for CVD. Other indices of carotid plaques, such as their properties, area, and thickness, might have had higher correlations with CVD. Therefore, additional research is needed to quantify the influence of carotid plaques on CVD outcomes. We need additional data to examine and evaluate the effect of carotid plaques on CVD. Second, we measured carotid artery plaques only once, at baseline, and the evolution of the plaques in time was not examined. Hypertension can cause atherosclerosis, which may interact with carotid plaques, especially plaques in the carotid sinus. Because of the location of the carotid sinus baroreceptor, plaques in the carotid sinus may have a larger effect on blood pressure regulation. We also need to examine the detection of carotid plaques in detail to improve methods of evaluating the risk for CVD events. Third, this study only considered diabetes based on a single FBG level at the 2010 to 2011 medical examination. An oral glucose tolerance test was not confirmed. This is likely to underestimate the risk of diabetes on cardiovascular and cerebrovascular diseases, but WHO believes that one‐time FBG results can be used in large‐scale epidemiological studies and many large‐scale epidemiological studies such as the Framingham study^39^, ^40^ used this method. The sample size of this study is large, and the participants did not move, resulting in minimal loss to follow‐up. The results still provide a scientific basis for the prevention and treatment of cardiovascular and cerebrovascular diseases.

## 5 CONCLUSIONS

In conclusion, we can improve our evaluations of levels of risk for CVD and all‐cause mortality, especially for stroke, with the use of ultrasound measurements in patients with combined hypertension and carotid plaques. This combined analysis was more sensitive to risk evaluations of CVD and all‐cause mortality than any single indicator.

## CONFLICT OF INTEREST

The authors declare no potential conflict of interests.

## ETHICS APPROVAL AND CONSENT TO PARTICIPATE

In this planned project, the participants' rights and interests were protected sufficiently, which satisfies the requirements of the World Medical Association's Declaration of Helsinki and the Medical Ethics Committee. This study protocol was approved prior to the study's commencement.

## Supporting information


**Table S1** Sensitivity analysis after removing the lipid‐lowering drugs

## Data Availability

All the data generated and analyzed during this study were included in this published article.
