# Supplementary material for: Combined effects of carotid plaques and hypertension on the risk of cardiovascular disease and all‐cause mortality
Source: Clin Cardiol. 2020 May 12;43(7):715–22. doi: 10.1002/clc.23372 (PMC7368304; doi:10.1002/clc.23372)
Supplement: Supplementary file 1 — Table S1 Sensitivity analysis after removing the lipid‐lowering drugs [file CLC-43-715-s001.doc]

**Table S1.** Sensitivity analysis after removing the lipid-lowering drugs

|  | No carotid plaque and normal blood pressure | Hypertension only | Carotid plaque only | Hypertension with carotid plaque | All | Log-rank |
| --- | --- | --- | --- | --- | --- | --- |
| All-cause mortality | 22 (0.99) | 24 (1.86) | 56 (4.94) | 108 (5.80) | 210 (3.23) | <0.01 |
| CVD | 19 (0.85) | 24 (1.86) | 26 (2.30) | 88 (4.73) | 157 (2.41) | <0.01 |
| Stroke | 11 (0.5) | 17 (1.3) | 20 (1.8) | 66 (3.5) | 114 (1.8) | <0.01 |
| Cerebral hemorrhage | 2 (0.09) | 1 (0.08) | 2 (0.18) | 3 (0.17) | 8 (0.14) | 0.82 |
| Cerebral ischemic | 9 (0.40) | 16 (1.24) | 18 (1.6) | 62 (3.30) | 105 (2.61) | <0.01 |
| MI | 8 (0.36) | 7 (0.54) | 6 (0.53) | 23 (1.24) | 44 (0.68) | 0.01 |

CVD: cardiovascular disease; MI: myocardial infarction.
